# Performance Evaluation and Community Application of Low-Cost Sensors for Ozone and Nitrogen Dioxide

**DOI:** 10.3390/s16101698

**Published:** 2016-10-13

**Authors:** Rachelle M. Duvall, Russell W. Long, Melinda R. Beaver, Keith G. Kronmiller, Michael L. Wheeler, James J. Szykman

**Affiliations:** 1 Office of Research and Development, U.S. Environmental Protection Agency, 109 T.W. Alexander Drive, Research Triangle Park, NC 27711, USA; long.russell@epa.gov (R.W.L.); james.j.szykman@nasa.gov (J.J.S.); 2 Office of Air Quality Planning and Standards, U.S. Environmental Protection Agency, 109 T.W. Alexander Drive, Research Triangle Park, NC 27711, USA; beaver.melinda@epa.gov; 3 Jacobs Technology Inc., 600 William Northern Boulevard, Tullahoma, TN 37388, USA; kronmiller.keith@epa.gov (K.G.K.); wheeler.michael@epa.gov (M.L.W.); 4 NASA Langley Research Center, 11 Langley Boulevard, Hampton, VA 23681, USA

**Keywords:** nitrogen dioxide, ozone, low-cost sensors, electrochemical sensor, performance evaluation, citizen science

## Abstract

This study reports on the performance of electrochemical-based low-cost sensors and their use in a community application. CairClip sensors were collocated with federal reference and equivalent methods and operated in a network of sites by citizen scientists (community members) in Houston, Texas and Denver, Colorado, under the umbrella of the NASA-led DISCOVER-AQ Earth Venture Mission. Measurements were focused on ozone (O_3_) and nitrogen dioxide (NO_2_). The performance evaluation showed that the CairClip O_3_/NO_2_ sensor provided a consistent measurement response to that of reference monitors (r^2^ = 0.79 in Houston; r^2^ = 0.72 in Denver) whereas the CairClip NO_2_ sensor measurements showed no agreement to reference measurements. The CairClip O_3_/NO_2_ sensor data from the citizen science sites compared favorably to measurements at nearby reference monitoring sites. This study provides important information on data quality from low-cost sensor technologies and is one of few studies that reports sensor data collected directly by citizen scientists.

## 1. Introduction

Under the United States Clean Air Act, nitrogen dioxide (NO_2_) and ozone (O_3_) are regulated as criteria pollutants, or commonly found air pollutants known to cause harmful effects on human health and the environment, as part of the National Ambient Air Quality Standards (NAAQS). These pollutants are routinely monitored by state and local agencies using Federal Reference Methods or Federal Equivalent Methods (FRM/FEM) for NAAQS compliance and other purposes [1]. A number of small, low-cost (~$100–$5,000 USD) sensor technologies for the measurement of criteria gases and other pollutants have recently emerged. These devices can provide near real-time, continuous measurements. Sensors have the potential for use in various applications such as outdoor and indoor air pollution monitoring, source or fence line monitoring, emissions inventory characterization, personal exposure monitoring, and community or individual monitoring activities [2,3,4]. Of these applications, community and individual monitoring has gained popularity as sensor devices are highly accessible, inexpensive compared to traditional air monitoring equipment, straightforward to use, portable, and have software, web interfaces, or smartphone applications to easily view and retrieve data. In addition, the public has a strong desire to know more about what air pollutants and corresponding levels they are being exposed to. Citizen science, which refers to public (citizens) involvement in “collecting, categorizing, transcribing, or analyzing scientific data” [5] is an example of community and individual monitoring. Citizen science can play an important role in augmenting scientific studies and non-regulatory monitoring by increasing the spatial coverage and time resolution of data, offering data for locations or groups that are adversely impacted by pollution, and helping to leverage resource burdens that are typically required for monitoring activities.

The performance of sensors has been an area of focus as there is still a need to determine the accuracy of data to support different applications of sensors. Numerous field and laboratory performance evaluations of low-cost sensors have been conducted. While many sensors have shown good performance in comparison to traditional regulatory monitoring equipment, there are still known issues with data quality and a need to understand long-term (12 months or more) sensor performance, performance in areas with poor air quality, cross interferences with other pollutants, and influences of temperature and relative humidity on the measurements [6,7,8,9,10,11,12,13,14,15]. Although these issues exist, air sensor technologies are quickly being adopted to measure air pollution, especially by individuals and communities who are eager to understand their exposures to air pollutants [2,3,4,16,17,18,19].

The goal of this study was to obtain information on the data quality of measurements from the CairClip O_3_/NO_2_ and CairClip NO_2_ sensors in real-world conditions. This study also sought to understand the feasibility of incorporating citizen science to expand spatial coverage of O_3_ and NO_2_ measurements. In this study the performance of electrochemical-based CairClip sensors was evaluated and the sensors were operated by citizen scientists in an ambient monitoring network during two month-long field campaigns under the umbrella of the NASA-led DISCOVER-AQ Earth Venture Mission [20]. The goal of DISCOVER-AQ was to understand how satellites can be used to better predict air quality near the earth’s surface using a combination of ground-based and aircraft measurements. Air quality measurements collected on the ground are critical for validating satellite measurements. The use of low-cost sensors combined with citizen science-led data collection can offer unique opportunities to supplement air quality monitoring locations for a host of applications. As such, gaining a better understanding of both the performance and accuracy of low-cost sensors and the elements needed to conduct an effective citizen science study is important.

## 2. Materials and Methods

### 2.1. Instrumentation

**Figure 1:**
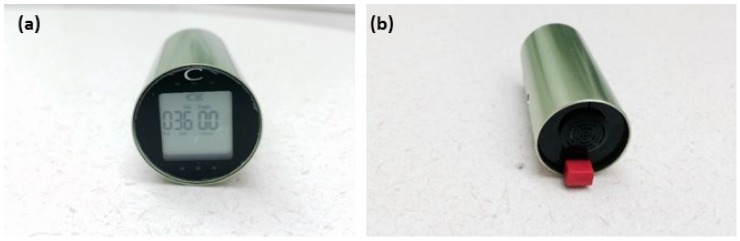
CairClip sensor front (**a**) and rear (**b**) views.

This study used the CairClip O_3_/NO_2_ and the CairClip NO_2_ sensors (Figure 1), manufactured by Cairpol (Alès, France). The CairClip O_3_/NO_2_ sensor provides a sum of O_3_ and NO_2_ (herein referred to as O_3_ + NO_2_) whereas the CairClip NO_2_ sensor measures NO_2_ only. The CairClip sensors were selected based on good performance relative to FRM/FEM analyzers (R^2^ > 0.99 and precision < 10 ppb) shown during a series of laboratory-based evaluations [10]. The CairClip sensor utilizes an electrochemical-based method to detect gaseous pollutants. The methodology behind this technique is briefly described here and more details can be found elsewhere [9,21]. Electrochemical sensors normally contain three electrodes: a working, reference and counter electrode. As a gas reaches the surface of the working electrode, it reacts and produces an electrical current between the working and counter electrode. The resulting current is linearly proportional to the gas concentration. The CairClip contains a micro fan, start/stop connecters, a particle air filter, and an internal data logging system. The sensors were calibrated by the manufacturer prior to receipt and no additional calibrations were performed before use. Data was retrieved using a USB interface and Cairsoft software (Cairpol). The CairClip was charged using either a USB cable connected to a computer or was continuously powered by a solar panel/battery system.

### 2.2. Measurement Sites During the Houston Campaign

#### 2.2.1. Performance Evaluation Site

One set of CairClip sensors (one NO_2_ and one O_3_/NO_2_) were collocated with Federal Reference Methods (FRMs) at the La Porte Airport (herein “LPA”; Latitude, Longitude = 29.672000, −95.064700) from 4–27 September 2013. Ozone was measured by an ethylene-chemiluminescence FRM using a Bendix Model 8002 analyzer. Nitrogen dioxide was measured by a gas-phase chemiluminescence FRM using a Teledyne Model T200U analyzer (Teledyne API; San Diego, CA, USA). The sensors were placed on the roof of the sampling trailer near the inlet of the FRM analyzers and housed inside an inverted aluminum bowl shelter to protect them from weather conditions. One-minute average data was obtained from both the FRM analyzers and the CairClip sensors.

#### 2.2.2. Citizen Science Sites

**Figure 2:**
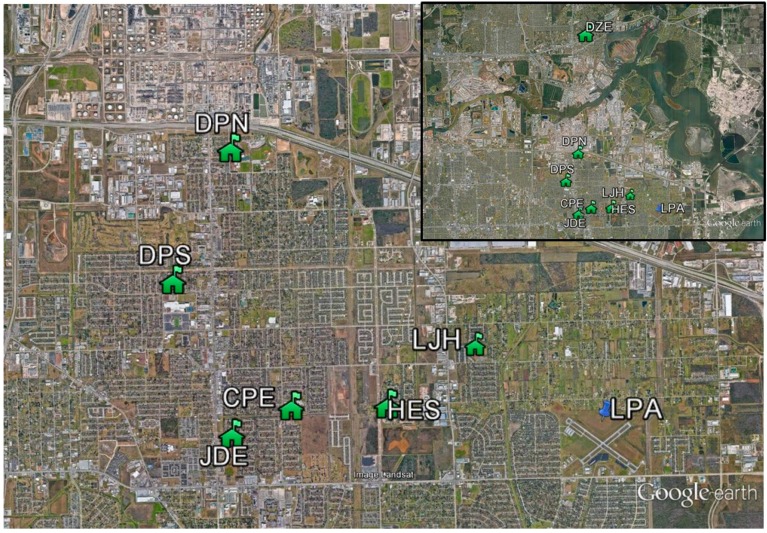
Map of the performance evaluation and citizen science sites in the Houston campaign.

One or two CairClip sensors were operated by citizen scientists (teachers and students) at seven schools throughout the Houston area from 4–27 September 2013. The schools were strategically selected to fall within the flight path of a NASA aircraft which collected air quality measurements in the study domain. The participating schools and CairClip versions used are provided in Figure 2 and Table 1. Four schools (College Park Elementary, JP Dabbs Elementary, Deer Park High School North, and DeZavala Elementary) ran only one CairClip sensor whereas the other three schools (Lomax Junior High, Heritage Elementary, and Deer Park High School South) ran one of each CairClip sensor version as shown in Table 1. With the assistance of science teachers, secure and easily accessible areas were identified for placement of sensors at each school. The sensors were housed inside an inverted aluminum bowl shelter to protect from weather conditions. The sensor housing was attached to a sturdy structure (e.g., flag poles, columns, or fences) at approximately 2.1 m above ground-level and placed away from high pollution point source areas (e.g., bus lines, student drop off/pick up areas). The citizens were trained on how to operate and setup the sensors and retrieve the data. The citizens deployed the sensors in the morning and collected them at the end of the day to prevent theft/damage and to recharge the sensors. The citizens decided which days to operate the sensors and were encouraged to collect measurements at minimum on days when the NASA aircraft would fly during the sampling campaign. The sensor performance evaluation site at LPA was used to assess the citizen science sensor data. One-minute average data was collected at all the sites.

### 2.3. Measurement Sites During the Denver Campaign

#### 2.3.1. Performance Evaluation Site

One set of CairClip sensors (one NO_2_ and one O_3_/NO_2_) were collocated with FRMs at a site in Golden, Colorado (herein “GOL”; Latitude, Longitude = 39.743725, −105.17799) from 14 July to 12 August 2014. Ozone was measured by a nitric oxide chemiluminescence FEM using a Teledyne Model T265 Analyzer (Teledyne API; San Diego, CA, USA). Nitrogen dioxide was measured by a gas-phase chemiluminescence FRM using a Teledyne Model T200U analyzer (Teledyne API; San Diego, CA, USA). The sensors were placed on the roof of the sampling trailer near the inlet of the FRM analyzers and housed inside a CairTub stainless steel enclosure (Cairpol) to protect from weather conditions. A solar power/battery system was attached to the CairTub to provide continuous power to the sensors. One-minute average data was obtained from the reference analyzers and CairClip sensors.

#### 2.3.2. Citizen Science Sites

**Figure 3:**
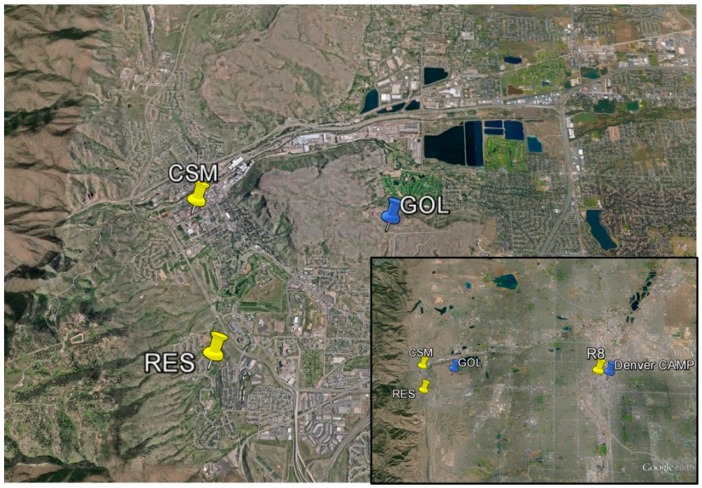
Map of the performance evaluation and citizen science sites in the Denver campaign.

One or two CairClip sensors were hosted by citizen scientists (community members) in the Denver area from 15 July to 12 August, 2014, at three locations including rooftops of a library at the Colorado School of Mines (CSM) and the EPA Region 8 Building (R8), and a local residence (RES), Figure 3. The CairClip versions operated at the citizen sites are shown in Table 1. Both versions of the CairClip were operated at the CSM and R8 and only one was operated at an RES as shown in Table 1. Citizen scientists were recruited via EPA Region 8 staff and through local contacts. With the assistance of citizen scientists, sensors were placed inside a CairTub enclosure which was attached to a weighted tripod at approximately 1.5 m above surface level. The units were placed in secure and easily accessible locations that were not directly downwind of high pollution areas. Sensors remained outdoors (24 h/7 days a week) and were continuously powered with a solar panel/battery system. Citizen scientists were shown how to operate the sensors and assisted with weekly data retrievals.

The sensor performance evaluation site (GOL) was used to assess the data collected at the CSM and RES citizen science sites. The R8 citizen science site data was compared to reference data from the nearby Denver CAMP state monitoring site (herein “CAMP”; Latitude, Longitude = 39.751184, −104.987625) which is maintained and operated by the Colorado Department of Public Health and the Environment. At the CAMP site, O_3_ was measured by an ultraviolet absorption FEM using a Teledyne Model 400E analyzer (Teledyne API; San Diego, CA, USA) and NO_2_ was measured by a gas-phase chemiluminescence FRM using a Teledyne Model T200U analyzer (Teledyne API; San Diego, CA, USA). One-minute average data was collected at all sites.

### 2.4. Data Analysis

Hourly averages and simple linear regression were calculated for the CairClip sensor and reference measurements using R statistical software, version 3.2.1 [22]. Separate O_3_ and NO_2_ values from the CairClip O_3_/NO_2_ sensor were obtained by subtracting the reference O_3_ and reference NO_2_ data, respectively, from the sensor data. The citizen science data was compared to the nearby reference monitoring sites. In the Houston campaign, sensors were not run continuously by the citizen scientists and the citizens did not routinely record when the sensors were operated versus not operated (turned on or off). In evaluating the data, it was difficult to distinguish the start/stop times, therefore, to avoid bias, linear regression analysis was not conducted on this data. Linear regression analysis was performed on the CairClip data from the citizen science sites in the Denver campaign as the data was collected continuously.

## 3. Results and Discussion

### 3.1. Performance Evaluation

Table 2 summarizes the hourly average CairClip sensor and reference measurements during the Houston (LPA site) and Denver (GOL site) campaigns. The CairClip NO_2_ sensor malfunctioned during the Denver campaign therefore no data is available. The CairClip O_3_/NO_2_ sensor consistently showed good agreement to reference measurements. Results from each campaign are described in more detail in the next sections.

#### 3.1.1. Houston Campaign

**Figure 4:**
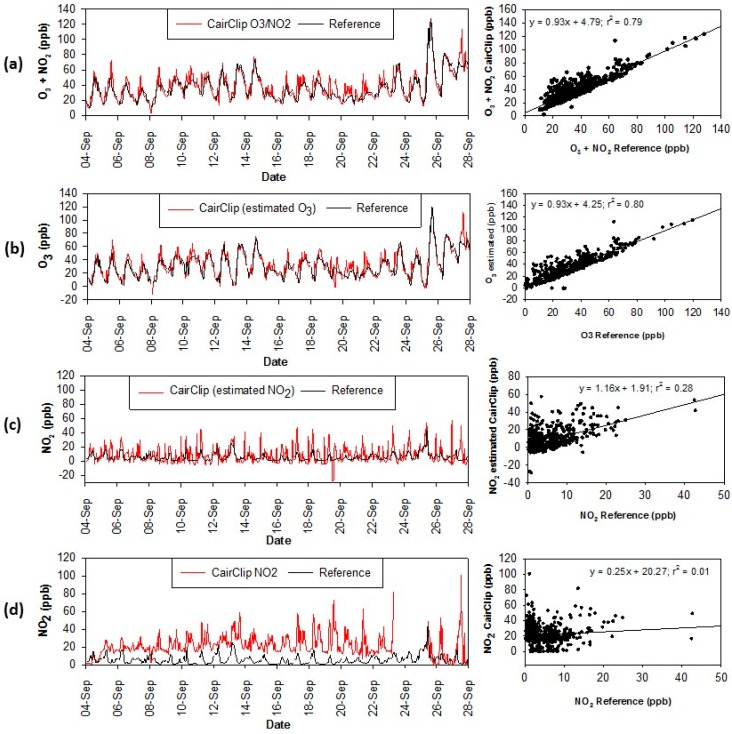
Time series and scatter plots of hourly average reference measurements during the Houston campaign compared to the (**a**) CairClip O_3_/NO_2_ sensor; (**b**) estimated O_3_ values for the CairClip O_3_/NO_2_ sensor; (**c**) estimated NO_2_ values for the CairClip O_3_/NO_2_ sensor; and (**d**) CairClip NO_2_ sensor.

A comparison of the hourly average concentrations from the CairClip O_3_/NO_2_ sensor and reference analyzers (summed O_3_ and NO_2_) is shown in Figure 4a. On average, the CairClip measurements were slightly higher than the reference measurements. Linear regression analysis indicated good correlation (r^2^ = 0.79) between the CairClip and reference O_3_ + NO_2_ data. In separating out the ozone measurements, good correlation was observed between the reference O_3_ data and estimated CairClip O_3_ values (Figure 4b, r^2^ = 0.80). The estimated CairClip NO_2_ values were higher than the reference measurements and minimal agreement was observed (Figure 4c, r^2^ = 0.28). This observation is consistent with another study reporting no correlation between calculated NO_2_ values from the CairClip sensor and reference NO_2_ data [11]. While the CairClip O_3_/NO_2_ sensor is designed to measure the sum of O_3_ and NO_2_, the sensor is inherently less sensitive to NO_2_ [7,10]. Hourly O_3_ levels were on average six times higher than NO_2_ levels in the Houston area during the study and likely dominated the measured CairClip values. The effects of temperature and humidity on the CairClip measurements were also assessed. This data was obtained from the local Houston Hobby Airport and showed moderate correlation with relative humidity (RH; r^2^ = 0.43) and slight correlation with temperature (r^2^ = 0.28; scatter plots and linear regression displayed in Appendix A).

The CairClip NO_2_ sensor data was more variable compared to the reference data (Figure 4d) and on average was significantly higher (21.4 ppb versus 5.5 ppb). No correlation (r^2^ < 0.10) was observed between the CairClip and reference measurements. The detection limit of the CairClip NO_2_ sensor is 20 ppb [8]. Given the low concentration of NO_2_ during the study (hourly average of 5.5 ppb) it is likely that the CairClip could not accurately detect NO_2_. The highest NO_2_ concentrations during the study were observed on 25 September 2013, which was an ozone exceedance day for the primary, 8 h NAAQS. The maximum NO_2_ concentration occurred at 11:00 Central Standard Time (CST) in which the reference analyzer and CairClip sensor reported 42.7 ppb and 49.2 ppb of NO_2_, respectively. This was one of the limited incidences where the CairClip value was within ±10 ppb of the reference value (occurred <25% of the time). Ozone is a known interference for NO_2_ electrochemical sensors. In addition, temperature and relative humidity can contribute to measurement biases. Due to the low NO_2_ levels throughout the study, these influences were not evaluated.

#### 3.1.2. Denver Campaign

**Figure 5:**
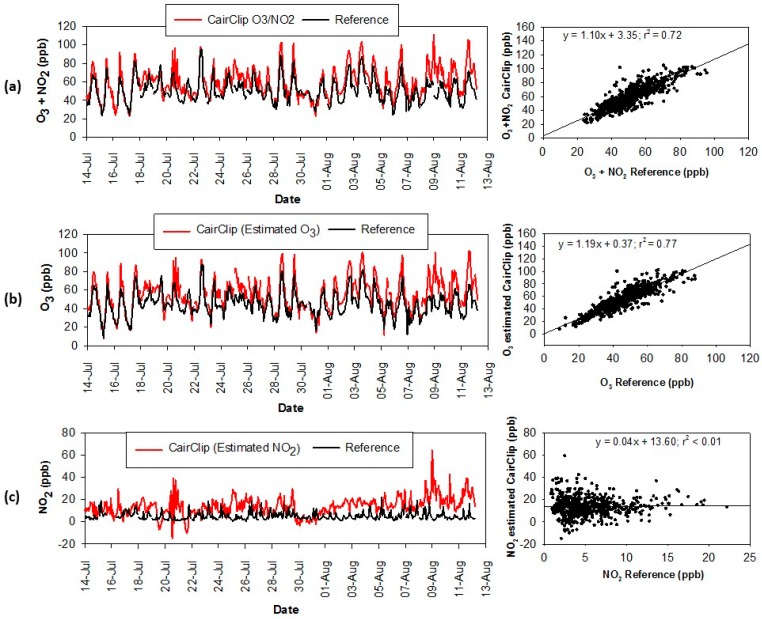
Time series and scatter plots of hourly average reference measurements during the Denver campaign compared to the (**a**) CairClip O_3_/NO_2_ sensor; (**b**) estimated O_3_ values for the CairClip O_3_/NO_2_ sensor; and (**c**) estimated NO_2_ values for the CairClip O_3_/NO_2_ sensor.

Hourly average concentrations from the CairClip O_3_/NO_2_ sensor and reference analyzers (summed O_3_ and NO_2_) are shown in Figure 5a and Table 2. While the CairClip NO_2_ sensor was co-located at the GOL site, it malfunctioned and the data was unavailable. The CairClip O_3_/NO_2_ sensor measurements on average were about 16% higher than the reference measurements. Overall, the CairClip O_3_/NO_2_ sensor showed good correlation with reference measurements (r^2^ = 0.72). Similar to the Houston campaign hourly average O_3_ levels were higher than NO_2_ levels (45.4 ppb versus 5.1 ppb) throughout the Denver campaign. Ozone and NO_2_ values from the CairClip O_3_/NO_2_ sensor were estimated similar to the method described for the Houston campaign. Good correlation was observed between the reference O_3_ data and estimated CairClip O_3_ values (Figure 5b, r^2^ = 0.77). The estimated CairClip NO_2_ values were higher than the reference data and no correlation was observed (Figure 5c, r^2^ < 0.01). Based on this data and the Houston data, estimating NO_2_ values from the CairClip O_3_/NO_2_ sensor may not provide true NO_2_ levels. The effects of temperature and RH (both measured at the GOL site) on the CairClip measurements were also evaluated and showed slight correlation (r^2^ = 0.34 for temperature and r^2^ = 0.31 for RH; scatter plots and linear regression analysis are displayed in Appendix A).

### 3.2. Citizen Science Application

The feasibility of sensors in citizen science-operated sites was evaluated. Overall, the citizen science data was comparable to the nearby sites containing reference measurements. Similar to the performance evaluation, the CairClip O_3_/NO_2_ sensor showed similar trends and better correlation with the reference data. Results from each sampling campaign are described in more detail in the next sections.

#### 3.2.1. Citizen Science Data from the Houston Campaign

**Figure 6:**
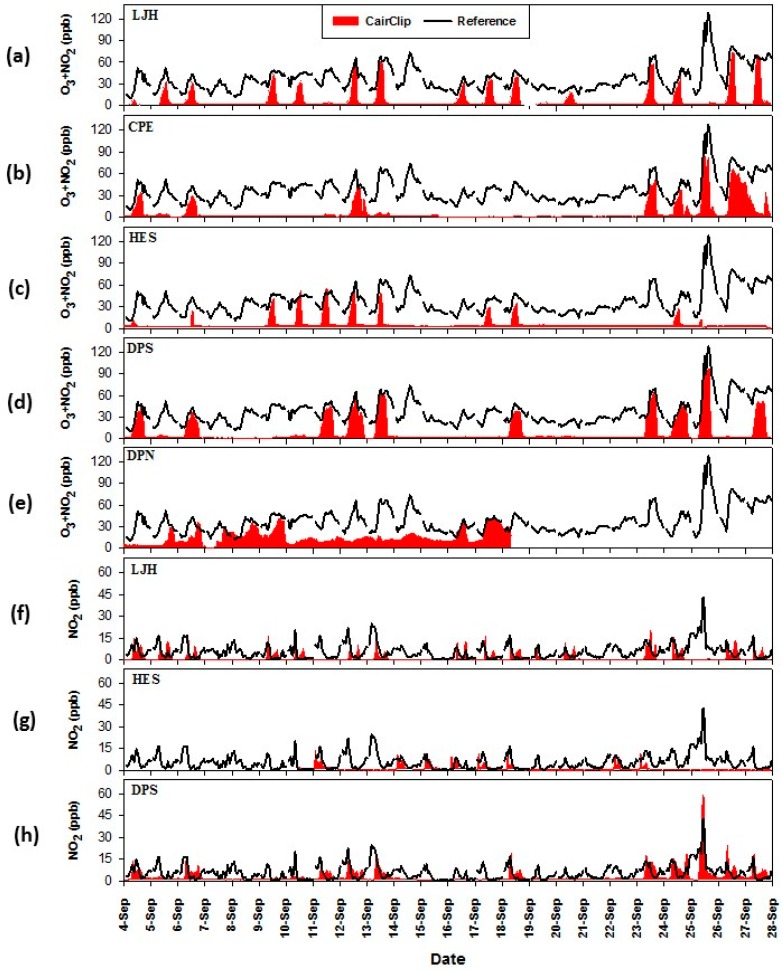
Measurements collected at the citizen science-operated sites in the Houston campaign for the (**a**) to (**e**) the CairClip O_3_/NO_2_ sensor and (**f**) to (**h**) the CairClip NO_2_ sensor.

Sampling days were spread out at each citizen science site. The sampling duration ranged from about 1 to 10 h between 07:00 and 20:00. Data from DeZavala Elementary (DZE) and JP Dabbs Elementary (JDE) was not available due to either sensor malfunction or data retrieval problems. The CairClip also malfunctioned at Deer Park High School North Campus (DPN) therefore only a partial data set was available. Hourly average CairClip O_3_/NO_2_ sensor data at the citizen science sites compared to the LPA reference data (summed O_3_ and NO_2_) is displayed in Figure 6a–e. CairClip values close to zero indicate times when the sensor was not operating. The citizen science CairClip data showed similar daily trends compared to the reference data. The highest hourly O_3_ + NO_2_ levels were observed at the Deer Park High South (DPS) and the College Park Elementary (CPE) citizen science sites on 25 September 2013 at 11:00. Hourly average concentrations from the CairClip O_3_/NO_2_ sensor at DPS, CPE and the reference analyzers at LPA were 99.4 ppb, 91.0 ppb, and 101.6 ppb, respectively. As mentioned, this day was a NAAQS ozone exceedance day for the primary, 8 h standard. Combined O_3_ and NO_2_ concentrations appeared to be fairly evenly distributed throughout the Houston area. Differences in concentration levels across the domain are anticipated due to local meteorology and atmospheric chemistry.

The CairClip NO_2_ sensor was operated at three citizen science sites and hourly average concentrations are shown in Figure 6f–h. It was difficult to distinguish clear trends between the CairClip and reference data. Nitrogen dioxide by nature is localized near emission sources and serves as a precursor for ground-level ozone formation. Therefore, it was expected that the NO_2_ concentrations measured in the Houston area would vary. The highest hourly NO_2_ concentration was observed at the DPS citizen science site on the ozone exceedance day (25 September 2013) at 10:00. On this day, the CairClip NO_2_ sensor at the DPS site and the LPA reference monitor reported 59.5 ppb and 42.4 ppb of NO_2_, respectively.

#### 3.2.2. Citizen Science Data from the Denver Campaign

**Figure 7:**
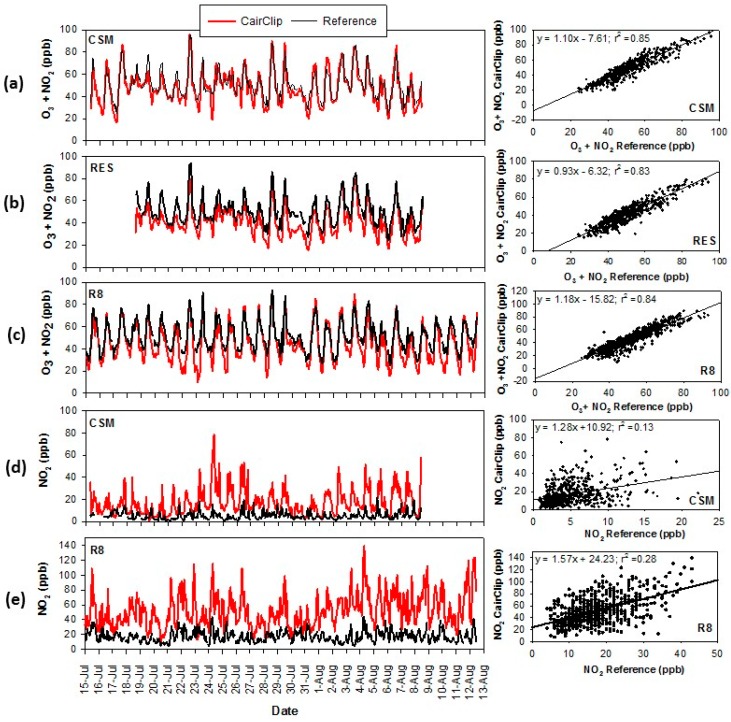
Measurements from the citizen science-operated sites compared to nearby reference data in the Denver campaign for the (**a**) to (**c**) CairClip O_3_/NO_2_ sensor and (**d**) to (**e**) CairClip NO_2_ sensor.

CairClip sensors were operated continuously by citizen scientists during the Denver campaign. Table 3 provides the hourly averaged CairClip and corresponding reference concentrations accompanied by the linear regression analysis results. Time series plots are shown in Figure 7. The CairClip O_3_/NO_2_ sensor measurements at the local residence (RES) and Colorado School of Mines (CSM) sites showed similar diurnal trends compared to the GOL reference data. The CSM site, closest to the GOL, showed the higher comparability. Similarly, at the R8 site the diurnal patterns compared favorably to the reference data from the Denver CAMP site. Average CairClip values ranged from 41.4 to 47.1 ppb at the citizen science sites. Good correlation between the CairClip sensor and reference data was observed at all the citizen science sites (r^2^ = 0.85 for the CSM; r^2^ = 0.83 for an RES; r^2^ = 0.84 for R8). Combined O_3_ and NO_2_ concentrations were evenly distributed throughout the Denver area.

The CairClip NO_2_ sensor consistently reported higher NO_2_ concentrations compared to the reference NO_2_ data (Table 3 and Figure 7). At the CSM and R8 sites, the diurnal patterns appeared to be similar to the reference data. Hourly average NO_2_ concentrations were the highest at the R8 citizen science site. This site was located in downtown Denver which has heavier traffic levels and thereby higher NO_2_ emissions. Minimal correlation was observed between the CairClip and reference measurements (r^2^ = 0.13 at the CSM; r^2^ = 0.28 at R8). The sensors at the CSM and R8 sites were located on rooftops of low- and mid-rise buildings, respectively. The elevation difference relative to the ground-based sites in addition to sensitivity of NO_2_ to local sources may have contributed to the differences observed between the sensor and reference measurements. The regression equations for these sites also showed large intercepts which may suggest interferences or influences by different pollutant sources and atmospheric processes.

### 3.3. Experiences with Citizen Scientists

Studies that incorporate citizen science can help better identify the types of information or procedures that are needed to ensure that high quality measurements are collected. All the citizen scientists participated on a voluntary basis. Specifically, for the Houston campaign, the schools used the activity for educational enrichment purposes. A number of lessons were learned throughout the study and recommendations for working with citizen scientists are provided in the next sections.

#### 3.3.1. Lessons Learned

The Houston campaign served as a pilot project for evaluating sensors and incorporating citizen scientists to assist in ambient data collection. Several lessons were learned during the Houston campaign that were implemented in the Denver campaign to improve data collection. First, the sensors in Houston were not operated continuously and in some instances only a few hours of data was collected. Although a data log was provided, the citizen scientists did not regularly document when the sensors were deployed and retrieved on a given day. In order to avoid bias in the statistical summaries from estimating start and stop times, we only used time series plots to investigate CairClip data comparability to the reference data. To remedy this in the Denver campaign, we used the CairTub sensor housing and attached a solar panel/battery system to charge the CairClip sensors and run the sensors continuously (24 h/7 days a week) at all the citizen science sites. We ultimately had a larger data set in which we were able to conduct regression analyses to investigate comparability of the citizen science and reference data. Secondly, the Houston sensor network was installed approximately two weeks before the official start date of the study. While science teachers at the schools were trained in person and received handouts containing detailed instructions on how to operate the sensors, the lag between training and sample collection made it difficult in some cases for the citizens to remember how to properly deploy or retrieve data from the sensor. As a result, some of the data was lost or not collected. During the Denver campaign, we installed the sensors with the help of the citizen scientists, provided a demo and instructional handout, and began data collection on the same day. Lastly, the start of the Houston campaign coincided with the beginning of a new school year. Some schools that initially agreed to host a sensor withdrew their participation as they were concerned about the time commitment involved. While schools were not in session during the Denver campaign, we were mindful of explaining to the citizen scientists the approximate time required to assist in data collection and retrieval.

#### 3.3.2. Recommendations for Collaborating with Citizen Scientists

Utilizing citizen scientists in studies such as DISCOVER-AQ can help augment a scientific study by adding data, expanding spatial coverage of data, and involving communities in real science. During both campaigns, the citizen scientists found the hands-on participation very educational and rewarding. Based on our experiences, we offer several recommendations for working with citizen scientists:

(1) Discuss the time commitment involved and expectations. Citizen scientists kindly offer their time to assist with a study, therefore it is important to clearly outline the time commitment that is expected of them. Based on this information they can decide whether or not they would like to participate in a study and know upfront how much time they need to devote to collecting measurements.
(2) Provide clear and easy-to-follow instructions for data collection. It is critical to provide instructions in laymen’s terms for citizen scientist groups who may be collecting an observation or operating equipment. These groups have a variety of backgrounds and experiences and may not necessarily be familiar with how to accurately capture and document a scientific observation. Providing a face-to-face demo, either in person or via voice over internet protocol applications (such as Skype) is ideal but one could also utilize other types of communications such as conference calls, webinars or instructional videos. Hand-outs should contain step-by-step, clearly labeled directions with pictures if available. Depending on the activity, the instructions should include details on how to operate and maintain a measurement device, how to collect data, and/or how to properly record information related to a measurement (e.g., outdoor weather conditions, irregular events). Providing clear instructions will help encourage collection of high quality data.
(3) Maintain frequent communication. Communicating with citizen scientists regularly ensures that questions or problems are addressed and that data collection runs smoothly. In some cases, citizens may have to download data from a measurement device and frequent communication can serve as a reminder to retrieve the data and/or conduct maintenance on a device.
(4) Interact with the citizen science community. Studies that include citizen science offer a unique opportunity to visit the citizens that are helping collect data as well as the local community. Citizens are often eager to learn about the studies they are contributing to and enjoy interacting with scientists. In particular, teachers find these opportunities invaluable in supplementing their curriculum and keeping students engaged in learning. During both DISCOVER-AQ campaigns, we presented hands-on science activities at schools and local community events reaching out to about 1500 individuals. These interactions were well-received and enjoyable for the community members.

## 4. Conclusions

In this research study, the performance evaluation of sensors showed that the CairClip O_3_/NO_2_ sensor had the highest agreement with reference measurements. Estimating O_3_ values from the CairClip O_3_/NO_2_ sensor showed good agreement to reference O_3_ measurements. However, estimating NO_2_ values from this sensor should be examined carefully as the data showed little to no agreement with reference NO_2_ data. The CairClip NO_2_ sensor showed little to no agreement with reference data likely, due to low NO_2_ levels during both field campaigns. This sensor requires additional evaluation, preferably in environments with consistently high NO_2_ concentration levels such as those found near roadways.

The citizen science-led data collection demonstrated that community participation in research studies can be used to complement data collected by experts. The CairClip O_3_/NO_2_ sensor data from the citizen science sites compared favorably to measurements at nearby reference monitoring sites. This data is anticipated to be used to support comparative analyses with other data collected during the DISCOVER-AQ Mission and to further examine spatial variability of O_3_ and NO_2_ across the study areas. Overall this work will inform research and potential applications in the evolving field of low-cost sensor technologies.

## Figures and Tables

**Table 1:** Citizen science and reference measurement sites.

Field Campaign	Site Name (Abbreviation)	City	Distance ^1^ (km)	CairClip Version
	Lomax Junior High School (LJH)	La Porte	3.2	NO_2_, O_3_/NO_2_
	Heritage Elementary School (HES)	La Porte	5.6	NO_2_, O_3_/NO_2_
	College Park Elementary School (CPE)	Deer Park	7.2	O_3_/NO_2_
**Houston, TX**	JP Dabbs Elementary School (JDE)	Deer Park	8.0	NO_2_
	Deer Park High School South (DPS)	Deer Park	9.0	NO_2_, O_3_/NO_2_
	Deer Park High School North (DPN)	Deer Park	9.7	O_3_/NO_2_
	DeZavala Elementary School (DZE)	Channelview	29.0	O_3_/NO_2_
	La Porte Airport Reference Site (LPA)	La Porte	-	NO_2_, O_3_/NO_2_
	Colorado School of Mines (CSM)	Golden	5.8	NO_2_, O_3_/NO_2_
	Local Residence (RES)	Golden	8.5	O_3_/NO_2_
**Denver, CO**	EPA Region 8 (R8)	Denver	1.8	NO_2_, O_3_/NO_2_
	Golden Reference Site (GOL)	Golden	-	NO_2_, O_3_/NO_2_
	Denver CAMP Reference Site (CAMP)	Denver	-	-

^1^ Distance from reference site.

**Table 2:** Measurements at the reference sites.

CairClip Version	Site Name	Sampling Days	Hourly Average (ppb) CairClip	Hourly Average (ppb) Reference	CairClip/Reference
O_3_/NO_2_	LPA	24	38.40	37.53 ^1^	1.02
	GOL	30	59.10	51.13 ^1^	1.16
NO_2_	LPA	24	21.45	5.54	3.87
	GOL	30	-	5.10	-

^1^ Sum of O_3_ and NO_2_.

**Table 3:** Measurements at the citizen science sites during the Denver campaign.

CairClip Version	Site	Sampling Days	Hourly Average (ppb) CairClip	Hourly Average (ppb) Reference	CairClip/Reference
O_3_/NO_2_	CSM	25	48.25	51.48 ^1^	0.94
	RES	22	41.33	51.48 ^1^	0.80
	R8	29	45.01	51.70 ^1^	0.87
NO_2_	CSM	25	17.01	5.14	3.31
	R8	29	51.79	17.29	3.00

^1^ Sum of O_3_ and NO_2_.

**Figure A1:**
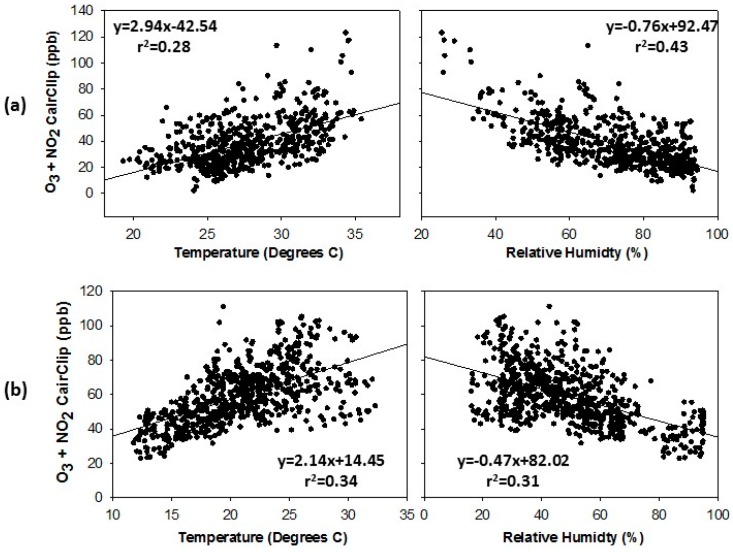
Scatter plots comparing CairClip O_3_/NO_2_ sensor measurements to temperature and relative humidity in (**a**) Houston campaign and (**b**) Denver campaign.
